# Pyroptosis in cardiovascular diseases: roles, mechanisms, and clinical implications

**DOI:** 10.3389/fcvm.2025.1629016

**Published:** 2025-08-04

**Authors:** Yuqing Niu, Li Wang, Yaoqing Zhang, Yanqiang Zou, Cheng Zhou

**Affiliations:** ^1^ Department of Cardiovascular Surgery, Union Hospital, Tongji Medical College, Huazhong University of Science and Technology, Wuhan, China; ^2^ Department of Cardiovascular Surgery, Jingshan Union Hospital, Union Hospital, Huazhong University of Science and Technology, Jingshan, Hubei, China; ^3^ Department of Pharmacy, Qiaokou District People’s Hospital, Wuhan, China

**Keywords:** pyroptosis, cardiovascular diseases, myocardial infarction, myocarditis, heart failure, atherosclerosis, hypertension, arrhythmia

## Abstract

Pyroptosis is an inflammatory form of programmed cell death, distinct from apoptosis, necroptosis, and ferroptosis, and is primarily mediated by gasdermin proteins and inflammatory caspases. Recent advances highlight the central role of pyroptosis in the pathogenesis and progression of a spectrum of cardiovascular diseases, including myocardial infarction, myocarditis, heart failure, atherosclerosis, hypertension, and cardiac arrhythmias. Activation of inflammasomes and the subsequent cleavage of gasdermins drive cell membrane pore formation, leading to the release of interleukin-1β (IL-1β), interleukin-18 (IL-18), and other pro-inflammatory mediators, amplifying tissue injury and sterile inflammation. Both experimental and clinical evidence reveal that targeting key molecules in the pyroptotic pathway, such as NLRP3 inflammasome, caspase-1, and gasdermin D, can attenuate myocardial injury, inhibit adverse cardiac remodeling, and stabilise atherosclerotic plaques. This review systematically summarises the current understanding of the molecular mechanisms of pyroptosis in cardiovascular pathology, details its disease-specific roles, and discusses translational and therapeutic perspectives. Modulating pyroptosis may provide new opportunities for the diagnosis, risk stratification, and treatment of cardiovascular diseases.

## 1 Introduction

Despite major advances in cardiovascular medicine, inflammation-driven cell death remains a fundamental challenge in the pathogenesis and progression of cardiovascular diseases (CVDs). While traditional forms of cell death such as apoptosis and necrosis have been extensively studied, the discovery of pyroptosis has unveiled a new dimension in inflammatory tissue injury. Pyroptosis is an inflammatory form of regulated cell death distinguished by gasdermin-mediated plasma membrane pore formation, cell swelling, and release of pro-inflammatory intracellular contents (1, 2). It is typically triggered by the activation of cytosolic pattern-recognition receptors (inflammasomes) in response to danger signals, leading to caspase-1 (canonical pathway) or caspase-4/5/11 (non-canonical pathway) activation and cleavage of gasdermin family effectors (3–6).

Pyroptosis has been shown to impact the development and progression of various CVDs, including myocardial infarction (MI), myocarditis, heart failure (HF), atherosclerosis, hypertension, and arrhythmias (7, 8). However, key questions persist regarding the triggers of pyroptosis in cardiac and vascular tissues, the interplay between different inflammasome pathways, and the clinical value of targeting pyroptosis as a therapeutic strategy. Additionally, the molecular distinction between pyroptosis and other cell death modalities in the cardiovascular context is not fully resolved. Addressing these gaps, this review summarises recent advances in our understanding of the molecular mechanisms of pyroptosis in cardiovascular pathology, highlighting key disease models and potential clinical implications. Notably, this review offers several novel insights beyond previous works. First, we integrate recent findings on non-canonical pyroptosis mediated by gasdermin E (GSDME), particularly in immune checkpoint inhibitor–induced myocarditis—an emerging and clinically relevant form of inflammatory cardiotoxicity. Second, we explore the contribution of pyroptosis to atrial arrhythmogenesis, including its impact on structural and electrical remodeling in atrial fibrillation, which remains under-recognized. Third, we provide a mechanistic overview of pyroptosis within broader inflammatory signaling frameworks such as PANoptosis and the cGAS–STING pathway. Finally, we discuss the clinical translation of pyroptosis-related biomarkers and therapies, highlighting current limitations and future opportunities. These elements collectively distinguish our review as a forward-looking synthesis that bridges molecular insights with potential clinical applications.

## 2 Myocardial infarction and ischaemic injury

### 2.1 Pathophysiological evidence

MI triggers an intense inflammatory reaction in the myocardium, and pyroptotic cell death has been strongly implicated in this process (9). In experimental models of myocardial ischaemia-reperfusion injury, cardiomyocyte pyroptosis is detected soon after reperfusion, contributing to infarct expansion and cardiac dysfunction (10). Genetic ablation of key pyroptosis mediators confers cardioprotection: mice lacking gasdermin D (GSDMD) have significantly reduced infarct sizes, less cardiomyocyte death, and improved post-MI cardiac function compared to wild-type mice (10–12). In a seminal study, GSDMD knockout in mice attenuated myocardial injury after coronary ligation, with fewer infiltrating neutrophils and macrophages in the infarct and reduced release of IL-1β (11). Correspondingly, pharmacological inhibition of caspase-1 with specific inhibitors (e.g., VX-765) during reperfusion limits infarct size and preserves left ventricular function in rodents (13). These findings indicate that the inflammasome-caspase-1-GSDMD pathway is activated by ischaemic injury and contributes to cardiomyocyte loss beyond apoptosis or necrosis (7). Mechanistically, I/R releases DAMPs including ATP, HMGB1, reactive oxygen species (ROS) that activate inflammasomes (especially NLRP3) in cardiomyocytes, macrophages, and infiltrating neutrophils (14, 15). The resulting pyroptotic cell death amplifies inflammation, creating a feed-forward cycle of injury. Clinically, patients with AMI have elevated circulating markers of pyroptosis. A study reported that plasma GSDMD levels are significantly higher in acute MI patients than in controls, correlating with infarct biomarkers and inflammatory cytokines (16). These human data support that pyroptosis is not merely a laboratory phenomenon but is active in human MI pathophysiology.

### 2.2 Molecular mechanisms

MI engages both canonical and non-canonical pyroptotic pathways. The canonical pathway is driven by inflammasome activation (chiefly NLRP3) and caspase-1 (17). In mouse models, myocardial I/R rapidly activates NLRP3 and caspase-1 in the heart, leading to IL-1β/IL-18 maturation and GSDMD cleavage in cardiomyocytes and resident immune cells (10). GSDMD N-terminal fragments form membrane pores, inducing osmotic lysis of cells and spillage of pro-inflammatory contents. Neutrophils have been shown to undergo pyroptosis in infarcted hearts, which exacerbates tissue injury; interestingly, neutrophil-derived proteases can also cleave GSDMD and contribute to IL-1β release (11). The non-canonical pathway involves caspase-11 (in mice; caspase-4/5 in humans) sensing intracellular LPS, which has relevance in sterile MI through gut microbial translocation or endogenous oxidised lipids acting similarly to activate caspase-11 (10, 18). Caspase-11 can induce pyroptosis by directly cleaving GSDMD and indirectly via NLRP3 activation. Indeed, one study showed that caspase-11 deficiency reduced infarct size, suggesting that non-canonical inflammasome activation contributes to myocardial I/R injury (10). Upstream, multiple inflammasome sensors may be involved: NLRP3 is the most studied and responds to mitochondrial ROS, Ca^2+^ flux, and ion imbalances during reperfusion (19, 20), but AIM2 (activated by DNA from necrotic cells) may also drive caspase-1 in MI, as suggested by elevated DNA-sensing pathway activation in infarct tissue (19, 21). Downstream, IL-1β and IL-18 released from pyroptotic cells act on surviving myocardium and infiltrating cells to augment inflammation and apoptotic pathways. IL-18, in particular, has been identified as a mediator of post-MI adverse remodeling; high serum IL-18 in MI patients predicts worse outcomes, linking pyroptosis to later HF (19). Thus, MI involves a complex interplay: ischaemia triggers inflammasome assembly (via NLRP3 and possibly others), caspase-1/-11 activation cleaves GSDMD, and pyroptosis of cardiomyocytes, neutrophils, and macrophages ensues, aggravating myocardial injury.

### 2.3 Clinical and therapeutic insights

Recognising pyroptosis as a driver of myocardial damage has spurred interest in anti-inflammatory therapies for MI. The IL-1β neutralising antibody canakinumab, tested in the CANTOS trial in post-MI patients, significantly reduced recurrent cardiovascular events, providing proof-of-concept that targeting inflammasome outputs benefits patients (22, 23). Although canakinumab's mechanism is broad IL-1β inhibition, its success underscores the pathological role of IL-1β (largely produced by inflammasome-pyroptosis activity) in post-infarction inflammation (24). Similarly, low-dose colchicine, an unspecific inflammasome inhibitor, lowered the risk of ischaemic events after MI in the COLCOT trial (25), hinting that suppressing inflammasome-driven inflammation (and by extension pyroptosis) is cardioprotective. These clinical trials align with animal studies where direct inhibition of inflammasome components or GSDMD has been beneficial. Selective NLRP3 inhibitors (like MCC950) have shown efficacy in reducing infarct size in preclinical MI models (porcine and murine) (26). Caspase-1 inhibitors (e.g., VX-765) improved cardiac function and reduced long-term remodeling when given at reperfusion in rodents (27–30). Such agents are now being explored for clinical use. Importantly, these interventions did not appear to compromise host defense acutely in sterile MI, suggesting a therapeutic window where modulating pyroptosis is beneficial. Beyond reducing acute injury, pyroptosis markers might serve as diagnostic or prognostic biomarkers in MI. As noted, circulating GSDMD or ASC specks have been proposed as indices of inflammasome activation (16), potentially aiding risk stratification. Moving forward, ongoing research aims to refine strategies to inhibit detrimental cardiac pyroptosis—for instance, using small-molecule gasdermin inhibitors or interfering with pyroptotic pore formation—to improve MI outcomes while avoiding undue immunosuppression. In summary, pyroptosis is a critical mediator of myocardial injury in infarction, and its modulation holds promise as a novel cardioprotective strategy.

## 3 Myocarditis

### 3.1 Evidence of involvement

Myocarditis, an inflammatory disease of the heart muscle often triggered by viral infection, has recently been linked to pyroptotic cell death as part of its pathogenic immune response. In Coxsackievirus B3 (CVB3)–induced viral myocarditis, myocardial tissues show activation of the NLRP3 inflammasome and increased caspase-1 activity, suggesting pyroptosis in infected cardiomyocytes and infiltrating immune cells (31, 32). A landmark study demonstrated that cathepsin B released during CVB3 infection can activate the NLRP3 inflammasome, leading to caspase-1–dependent pyroptosis and worsening myocardial injury. In mice, pharmacologic inhibition or genetic deletion of cathepsin B markedly reduced caspase-1 activation and IL-1β release in the heart, thereby attenuating myocarditis severity (31, 33). This indicates that Recent evidence implicates is a key mechanism by which enteroviral infection causes cardiomyocyte death and inflammation. Furthermore, IL-1β and IL-18 levels are elevated in myocarditis, implicating inflammasome activation; indeed, myocardial biopsies from myocarditis patients have shown increased NLRP3 and IL-1β expression (32). Beyond infectious causes, immune checkpoint inhibitor (ICI) therapy used in cancer can induce fulminant autoimmune myocarditis (34). Recent evidence implicates pyroptosis in ICI myocarditis: a 2024 study found that gasdermin E (GSDME) –mediated pyroptosis (rather than GSDMD) is extensively activated in ICI-related myocarditis, both in a mouse model and in patient heart samples (35). Mice lacking GSDME were protected from ICI myocarditis, with less immune cell infiltration and improved survival, demonstrating a direct pathogenic role for pyroptosis in this setting. Together, these findings across viral and immune-mediated myocarditis establish that pyroptosis contributes to cardiomyocyte loss and inflammatory amplification in the myocarditic heart.

### 3.2 Molecular mechanisms

In viral myocarditis, the interplay between viral pathogen-associated molecular patterns (PAMPs) and host sensors drives pyroptosis (36). Enteroviruses like CVB3 cause cardiomyocyte damage that releases cathepsin B from lysosomes; cathepsin B in the cytosol can trigger NLRP3 inflammasome assembly, perhaps by promoting mitochondrial dysfunction and reactive oxygen species. NLRP3 then activates caspase-1, leading to GSDMD pore formation and pyroptosis of infected cells. This not only kills cardiomyocytes, exacerbating ventricular dysfunction but also unleashes IL-1β/IL-18 which recruit and activate immune cells, fueling a vicious cycle of myocardium-targeted inflammation. Supporting this, interventions like IL-1 blockade or NLRP3 inhibition ameliorate experimental myocarditis (32). Another mechanism involves alarmins from necrotic cells: e.g., DNA from damaged cardiomyocytes can activate AIM2 inflammasomes in macrophages, potentially contributing to pyroptosis and cytokine release in myocarditis. In ICI-induced myocarditis, hyperactivated T cells produce excessive IFN-γ, triggering caspase-3 activation in cardiomyocytes, which cleaves GSDME to execute pyroptosis via pore formation (37–39). GSDME-mediated mitochondrial DNA release activates the cGAS-STING pathway, amplifying IFN and inflammatory responses through a feed-forward loop (35, 40, 41). This highlights non-canonical pyroptosis pathways (caspase-3/GSDME) in disease pathogenesis. Elevated IL-1β drives myocardial inflammation, with IL-1 neutralization improving outcomes, while IL-18 contributes to systemic symptoms (42). Pyroptosis coexists with apoptosis and necroptosis, forming PANoptosis in fulminant myocarditis (31, 43). Inflammasome activation and gasdermin pore formation remain central to myocardial injury, underscoring their therapeutic targeting potential.

**Figure 1:**
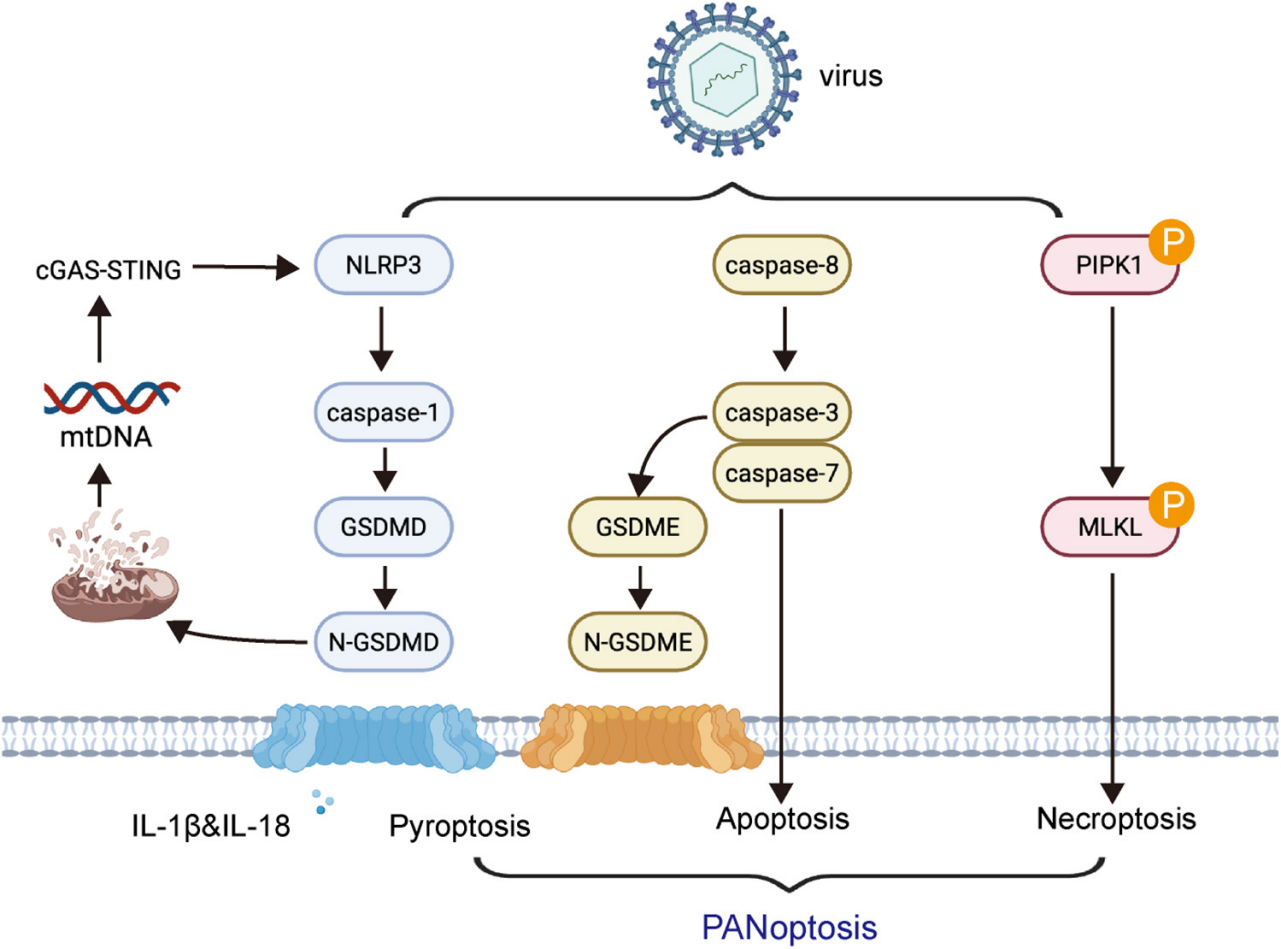
Mechanistic interplay of pyroptosis with other cell death pathways and immune signaling in myocarditis. Upon viral infection such as coxsackievirus B3 (CVB3), intracellular viral sensors including ZBP1 and RIG-I are activated in cardiomyocytes. These sensors initiate the assembly of the PANoptosome, which orchestrates the simultaneous activation of pyroptosis (*via* caspase-1 and GSDMD), apoptosis (*via* caspase-3), and necroptosis (*via* RIPK3 and MLKL). Gasdermin D (GSDMD)-mediated membrane pore formation facilitates the release of mitochondrial DNA (mtDNA) into the cytosol, which triggers the cGAS–STING signaling pathway.

Recent studies have demonstrated that viral myocarditis involves PANoptosis-dependent cell death pathways, wherein pyroptosis, apoptosis, and necroptosis are activated in a coordinated manner (43). Upon viral infection—particularly with coxsackievirus B3 (CVB3)—cytosolic viral sensors such as Z-DNA-binding protein 1 (ZBP1) and RIG-I are upregulated in cardiomyocytes. These sensors promote the formation of the PANoptosome complex, a multiprotein platform that orchestrates the activation of caspase-1 (pyroptosis via GSDMD), caspase-3 (apoptosis), and RIPK3/MLKL (necroptosis) (44). Among these, GSDMD-mediated pyroptosis plays a central role not only by causing membrane rupture but also by facilitating the release of mitochondrial DNA (mtDNA) into the cytosol (35). This mtDNA activates the cGAS–STING pathway, leading to type I interferon signaling and amplifying innate immune responses (45). This mechanistic interplay between pyroptosis, other cell death pathways, and cGAS–STING signaling forms a feed-forward inflammatory loop that drives myocardial injury in viral myocarditis (Figure 1).

### 3.3 Clinical implications

Understanding the role of pyroptosis in myocarditis opens avenues for targeted therapy and better biomarkers. Clinically, myocarditis ranges from mild to life-threatening, and current treatments are mainly supportive or immunosuppressive (for giant-cell or immune myocarditis). The evidence that IL-1 plays a causal role (via pyroptosis) has spurred trials of anakinra (an IL-1 receptor antagonist) in acute myocarditis (46). Case reports and small series have noted rapid improvement in severe myocarditis with anakinra, aligning with pyroptosis's pathogenic role. Likewise, NLRP3 inhibitors or caspase-1 inhibitors might attenuate myocardial inflammation—experimental IL-37 therapy (a cytokine that broadly suppresses inflammasome activity) dramatically reduced cardiac inflammation and improved survival in CVB3 myocarditis mice, highlighting inflammasome inhibition as a potential strategy (32, 47). For ICI-myocarditis, recognizing pyroptosis involvement suggests that adding inflammasome or gasdermin inhibitors to immunosuppressive regimens might better protect the heart while allowing some anti-tumor immunity to continue (35). Indeed, in the preclinical study, a small-molecule GSDME inhibitor reduced cardiac damage without entirely abrogating the immune response. In terms of diagnosis, endomyocardial biopsy showing active caspase-1 or GSDMD pores could help confirm myocarditis and distinguish it from ischaemic injury (35, 48). Additionally, circulating IL-18 or even cardiac troponin combined with IL-1 could improve diagnostic specificity for myocarditis if validated. An important translational insight is that therapies targeting pyroptosis (e.g., NLRP3 inhibitors like dapansutrile) are already in trials for other inflammatory diseases and could be repurposed for myocarditis. Overall, pyroptosis represents a novel therapeutic target in myocarditis: by dampening the inflammasome-gasdermin axis, one might quell the hyperinflammatory myocardial milieu, reduce tissue destruction, and preserve cardiac function in affected patients.

## 4 Heart failure and cardiac remodeling

### 4.1 Pyroptosis in heart failure pathophysiology

HF, whether secondary to ischaemic injury or chronic pressure/volume overload, is characterised by the progressive loss of cardiomyocytes, fibrotic remodeling, and ongoing inflammation (49, 50). Recent studies reveal that pyroptosis significantly contributes to cardiomyocyte loss and adverse cardiac remodeling in HF (51–53). In non-ischaemic dilated cardiomyopathy, myocardial tissues display increased NLRP3, cleaved caspase-1, and GSDMD expression compared to controls, consistent with persistent pyroptotic activity (52). Animal models further confirm this: NLRP3 activation in cardiomyocytes drives pyroptosis and promotes HF progression, while genetic silencing of NLRP3 or pharmacologic inhibition of caspase-1 improves ventricular function and reduces fibrosis (10). Pressure overload, as seen in hypertension or aortic stenosis, is another key cause of HF in which pyroptosis plays a pathogenic role. In mouse models with transaortic constriction (TAC), cardiomyocyte membrane rupture and IL-1β release—a hallmark of pyroptosis—have been observed. Inhibition of caspase-1 or knockout of NLRP3 protects against hypertrophy and cardiac dysfunction, whereas NLRP3 overexpression exacerbates hypertrophy under stress (54). These findings underscore that chronic cardiac stress leads to inflammasome activation and pyroptotic cardiomyocyte death, thus advancing HF. Clinically, chronic HF patients, particularly those with prior MI or diabetic cardiomyopathy, exhibit elevated circulating IL-1β and IL-18. Myocardial biopsies from end-stage HF have demonstrated active caspase-1 and inflammasome components, supporting the notion of ongoing, low-level pyroptosis that contributes to HF progression by persistent cell death and sterile myocardial inflammation.

### 4.2 Mechanistic insights

Multiple triggers induce pyroptosis in chronic HF. In pressure overload-induced HF, myocardial stretch and neurohormonal activation (angiotensin II, catecholamines) promote oxidative stress and mitochondrial dysfunction in cardiomyocytes—potent activators of the NLRP3 inflammasome. Once activated, the NLRP3/caspase-1 pathway induces release of IL-1β and IL-18, both with critical effects in HF: IL-1β impairs contractility and stimulates fibroblast activation, while IL-18 promotes myocyte hypertrophy and amplifies inflammation. Elevated IL-18 levels have been found in hypertrophic and failing hearts; mice lacking IL-18 are less susceptible to pressure-overload-induced hypertrophy, implicating pyroptosis-derived IL-18 in pathological hypertrophy (55). In metabolic or diabetic cardiomyopathy, factors such as high glucose, free fatty acids, and ceramides can also activate inflammasomes in cardiac cells (56, 57). For example, hyperglycaemia-induced ROS activate NLRP3, leading to cardiomyocyte pyroptosis and contractile dysfunction. Moreover, non-myocyte cardiac cells—including macrophages and fibroblasts—can also undergo inflammasome activation, contributing to adverse remodeling. Activated fibroblasts release IL-1, further weakening myocardial tissue. Pyroptosis in endothelial and smooth muscle cells of the cardiac microvasculature may worsen HF by compromising microcirculation and inducing cytokine production, though this is less well studied.

At the molecular level, a feed-forward loop often operates in HF: initial cell death from infarction or stress releases DAMPs (e.g., ATP, DNA), which in turn activate inflammasomes in neighbouring cells, promoting further pyroptosis and DAMP release (58). This perpetuates a chronic inflammatory state in the failing heart. Additionally, pyroptosis and apoptosis are interconnected. Caspase-8, traditionally apoptotic, can promote IL-1 production via NLRP3, while caspase-3 can cleave GSDME to trigger secondary pyroptosis—especially relevant in advanced HF with ischaemic episodes. Thus, once cell death pathways are activated in HF, pyroptosis can become a major mechanism of cell loss.

### 4.3 Therapeutic perspectives

The role of pyroptosis in HF highlights promising therapeutic targets. Several interventions have been investigated in preclinical and early clinical studies. Anti-IL-1 therapies have shown benefit: in acute decompensated HF, anakinra improved exercise tolerance and reduced inflammation, likely reflecting reduced pyroptosis-driven cytokine production (59). Colchicine, a broad anti-inflammatory agent, is being tested for its potential to limit cardiac remodeling by inhibiting inflammasome activity (60). Targeted drugs, such as dapansutrile (an NLRP3 inhibitor), have demonstrated reduction in inflammatory markers and improved diastolic function in HF with preserved ejection fraction (61). Direct inhibition of GSDMD offers another avenue—selective inhibitors can block pore formation, preventing pyroptosis regardless of upstream triggers (62). Preclinical studies show that GSDMD inhibition can reduce fibrosis and improve ejection fraction in HF models without major immunosuppression.

Pyroptosis pathway components may also serve as biomarkers in HF. Elevated IL-18 correlates with poor prognosis and ventricular dysfunction and could help identify patients with high inflammasome activity. ASC specks, oligomers derived from inflammasomes, have been detected in the circulation in inflammatory conditions and might indicate active cardiac inflammasomes if found in HF patients (63). Importantly, some inflammation is necessary for myocardial repair, so future therapies must carefully target pathological, not adaptive, pyroptosis. Research into cardioprotective factors such as irisin—a myokine upregulated by exercise—shows potential in inhibiting cardiac NLRP3 inflammasomes and pyroptosis, suggesting that metabolic interventions could also modulate pyroptosis.

In summary, pyroptosis is a significant driver of inflammation and cell loss in HF. Targeting this pathway could slow HF progression, improve cardiac function, and inform both prognosis and therapy in clinical practice.

## 5 Atherosclerosis

### 5.1 Role in atherosclerotic plaque development

Atherosclerosis is fundamentally an inflammatory disease of the arteries. Recent research has established pyroptosis as a critical mechanism linking cholesterol-induced metabolic stress to arterial inflammation and plaque development (64–67). During early atherogenesis, cholesterol crystals and oxidised LDL within the arterial wall serve as danger-associated molecular patterns (DAMPs), activating the NLRP3 inflammasome in macrophages (64, 68). This triggers caspase-1 activation and the release of IL-1β and IL-18, key cytokines that amplify local inflammation and recruit additional immune cells. Duewell et al. first showed that cholesterol crystals stimulate NLRP3-dependent IL-1β release, directly promoting atherosclerosis (69). Pyroptotic macrophage death within plaques contributes to the formation of necrotic cores—regions filled with debris and extracellular lipid—which destabilise plaque structure. Studies have detected active GSDMD and increased IL-1β secretion in atherosclerotic lesions, supporting the presence of ongoing macrophage pyroptosis within plaques (67, 70, 71). The significance of IL-1β from pyroptosis is underscored by genetic studies: ApoE-deficient mice lacking IL-1β show a marked reduction in lesion size, demonstrating the pivotal role of IL-1β in plaque growth (70). Conversely, deficiency of the IL-1 receptor antagonist accelerates atherosclerosis, emphasising that unchecked IL-1 signalling, much of it stemming from pyroptosis, exacerbates disease (72). IL-18, although its role is more nuanced, also appears to foster plaque progression and instability. Thus, pyroptosis fuels a vicious cycle within plaques: as macrophages ingest excess lipids and become foam cells, sustained cholesterol overload activates inflammasomes, leading to pyroptotic foam cell death and the release of cellular contents (lipids, enzymes, cytokines) that intensify inflammation and necrotic core expansion.

### 5.2 Mechanisms and cell types

Multiple cell types within atherosclerotic lesions can undergo pyroptosis, with macrophages being the most prominent. Foam cells—lipid-laden macrophages—struggle with cholesterol clearance, resulting in cholesterol crystal accumulation and persistent NLRP3 activation. The pyroptotic death of these macrophages not only releases IL-1β, but also acts on endothelial and vascular smooth muscle cells (VSMCs). In VSMCs, IL-1β and IL-18 induce adhesion molecules and chemokines, attracting more monocytes into plaques (73). IL-1β can also suppress collagen synthesis in VSMCs, potentially thinning the fibrous cap and increasing the risk of plaque rupture. While IL-1β deficiency reduces plaque burden, it is also associated with thicker fibrous caps, hinting that IL-1β influences plaque composition and stability (73). VSMCs themselves can activate inflammasomes under oxidative or metabolic stress; VSMC pyroptosis has been linked to vascular calcification in advanced plaques, partly through the release of matrix vesicles. Endothelial cells are also susceptible: exposure to disturbed flow or oxidised LDL can trigger inflammasome activation and local IL-1β release, promoting endothelial pyroptosis, dysfunction, and increased permeability to lipids (74). Notably, there is crosstalk between apoptotic and pyroptotic pathways in plaques. Macrophages initially undergoing apoptosis due to ER stress may progress to pyroptosis if caspase-1 is activated—a process called apoptosis-associated speck-like protein containing a CARD (ASC)-mediated pyroptosis. This leads to abundant inflammasome activation in cholesterol- and cell debris-rich lesions.

A hallmark of advanced plaques is the necrotic core, comprising remnants of numerous pyroptotic macrophages. This region is highly pro-thrombotic and destabilises plaques, making them prone to rupture and clinical events. Pyroptosis is believed to enlarge the necrotic core by causing rapid foam cell lysis and the release of prothrombotic factors such as tissue factor. Additionally, IL-18 from pyroptotic cells can induce apoptosis in surrounding VSMCs, further weakening plaque structure. In contrast, controlled, non-inflammatory apoptosis of macrophages can benefit early plaque regression; however, pyroptosis shifts this balance toward inflammation and instability. In essence, pyroptosis transforms relatively stable lipid storage into highly inflammatory, rupture-prone plaques.

### 5.3 Clinical and translational insights

Recognition of the inflammasome–pyroptosis–IL-1β axis has shaped new therapeutic strategies. The CANTOS trial using canakinumab, an IL-1β inhibitor, was the first to demonstrate that blocking this cytokine can significantly reduce major cardiovascular events in patients with prior MI and elevated inflammation (22, 73). This finding strongly implicates inflammasome-driven IL-1β—mainly from plaque macrophages—in atherogenesis and its complications. While canakinumab targets IL-1β broadly and not specifically pyroptosis, its success has spurred interest in upstream interventions. Colchicine, which suppresses inflammasome assembly and activity, has also shown efficacy in reducing cardiovascular events in patients with chronic coronary disease and post-MI (75). Experimental NLRP3 inhibitors, such as MCC950, have shown promise in preclinical models: in hyperlipidaemic mice, MCC950 reduces plaque size and complexity, at least partly by preventing macrophage pyroptosis and necrotic core formation (70). This suggests that therapies targeting NLRP3 may stabilise plaques by preserving macrophage viability or shifting cell death toward less inflammatory mechanisms. Therapies that enhance cholesterol efflux (e.g., HDL mimetics) may also reduce pyroptosis by alleviating cholesterol crystal burden in foam cells.

Biomarker development is ongoing. Elevated IL-18 levels are associated with higher cardiovascular risk and may help identify patients likely to benefit from anti-IL-1 or anti-inflammatory therapy (76). Measurement of GSDMD or caspase-1 activity in blood cells might serve as indicators of systemic inflammasome activation in atherosclerosis, though this remains investigational. Importantly, not all inflammasome activity is harmful: some studies suggest that complete NLRP3 deficiency does not always decrease atherosclerosis, possibly due to compensation by alternative inflammatory pathways. Thus, patient selection and combination therapy may be necessary. Another relevant consideration is the role of infectious agents. Pathogens such as periodontal bacteria have been linked to NLRP3 activation in arteries, implying that infection control may indirectly reduce pyroptosis within plaques (77).

In conclusion, pyroptosis is a central process in the formation and destabilisation of atherosclerotic lesions. Targeting the inflammasome–pyroptosis pathway—*via* inhibition of upstream triggers, key proteins (NLRP3, caspase-1, GSDMD), or downstream cytokines (IL-1β, IL-18)—represents a promising strategy to prevent or treat atherosclerotic CVDs.

## 6 Hypertension

### 6.1 Inflammatory paradigm and pyroptosis in hypertension

Hypertension is the most prevalent modifiable risk factor for cardiovascular morbidity and mortality worldwide, classically defined by a persistent elevation in systolic and/or diastolic blood pressure (78). Accumulating evidence has established chronic low-grade inflammation as a central contributor to the development and maintenance of hypertension, with increased infiltration of immune cells and elevated pro-inflammatory cytokines detectable in both hypertensive patients and animal models (53, 79). Among these, IL-1β and IL-18 are consistently elevated in essential hypertension and act as key mediators of vascular inflammation and end-organ damage (53). Recent studies have confirmed overactivation of the inflammasome and pyroptotic pathways in the cardiovascular and renal complications associated with hypertension. For example, downregulation or pharmacological inhibition of key inflammasome components (such as NLRP3 or caspase-1) markedly attenuates blood pressure elevation in various hypertensive animal models (80). Meanwhile, research with two PH rat models and hypoxic human pulmonary arterial smooth muscle cells (hPASMCs) indicates that pyroptosis contributes to pulmonary vascular fibrosis in pulmonary hypertension, with caspase-1 activation and STAT1-mediated PD-L1 upregulation in smooth muscle cells playing key roles in disease progression (81). Thus, elucidating the role of pyroptosis in hypertension pathogenesis not only provides mechanistic insight but also offers novel therapeutic opportunities.

### 6.2 Molecular mechanisms linking hypertensive stimuli to pyroptosis

Common hypertensive stimuli—including elevated angiotensin II (Ang II) and high dietary salt—elicit excessive production of ROS. ROS triggers dissociation of thioredoxin-interacting protein (TXNIP), which subsequently binds and activates the NLRP3 inflammasome (82). Sustained activation of this Ang II/ROS/TXNIP/NLRP3 axis in hypertensive states drives pyroptotic cell death and robust local inflammation in target organs such as the heart, vasculature, kidney, and brain (82). *In vitro* studies demonstrate that Ang II exposure induces NLRP3 activation and IL-1β production in tubular epithelial cells in a dose- and time-dependent manner, which can be mitigated by ROS scavenging or NLRP3 knockdown (83). These data collectively support the concept that pyroptosis is a key intermediary between classic hypertensive insults and subsequent target organ damage (82).

### 6.3 Pyroptosis in cardiovascular and renal target organ damage

Renal involvement is a hallmark of hypertension-related end-organ damage. Ang II and high salt exposure induce mitochondrial dysfunction and excessive ROS production in renal tubular epithelial cells, activating NLRP3 and promoting pyroptotic cell death (83). In murine models, NLRP3 knockout protects against tubular injury and proteinuria during chronic Ang II infusion, confirming the pathogenic role of the inflammasome in hypertensive nephropathy (83). Salt-sensitive hypertension models (such as 1 K/DOCA/salt mice and Dahl salt-sensitive rats) display marked activation of renal NLRP3 inflammasome and IL-1β production, which are attenuated by genetic or pharmacological inhibition of NLRP3 (e.g., MCC950) (80, 84). Notably, blockade of IL-1 signalling with anakinra in such models significantly reduces blood pressure and renal fibrosis, further substantiating the role of IL-1β as a mediator of hypertensive renal injury (85).

### 6.4 Central nervous system inflammasome activation

Emerging evidence indicates that the central nervous system, particularly key regulatory nuclei within the hypothalamus, is susceptible to inflammasome activation in hypertension. Rodent studies show that high-salt diets induce activation of microglia and NLRP3 inflammasome in the hypothalamic paraventricular nucleus (PVN), promoting neuroinflammation and heightened sympathetic outflow (86). Selective blockade of NLRP3 within the PVN dampens local inflammatory responses, attenuates sympathetic excitation, and delays blood pressure elevation (86). While these findings are mainly limited to animal studies, they provide mechanistic insight into the contribution of central neuro-inflammation to hypertension and suggest new potential targets for intervention.

### 6.5 Translational and therapeutic perspectives

Given the central role of pyroptosis in hypertensive target organ damage, targeting the inflammasome–pyroptosis axis represents a promising therapeutic avenue. Preclinical studies have demonstrated that pharmacological inhibition of the NLRP3 inflammasome (e.g., MCC950) effectively reduces blood pressure and attenuates cardiac and renal injury in a variety of hypertensive models (80). Similarly, IL-1 receptor antagonists such as anakinra confer blood pressure-lowering and organ-protective effects (85). While broad-spectrum anti-inflammatory agents like colchicine have shown efficacy in reducing cardiovascular events in coronary artery disease, their ability to inhibit inflammasome assembly provides a rationale for their ongoing evaluation in hypertension (25, 87). Notably, monoclonal antibodies targeting IL-1β (such as canakinumab) have reduced recurrent cardiovascular events in large clinical trials, despite having little direct effect on blood pressure, suggesting that inflammasome inhibition may improve hypertension-related outcomes even in the absence of antihypertensive effects *per se* (22).

In summary, the evidence to date underscores pyroptosis as a pivotal link between classical hypertensive stimuli and downstream end-organ damage. Targeting this pathway holds promise for the dual aims of blood pressure reduction and organ protection, ushering in a potential paradigm shift towards integrated “anti-hypertensive plus anti-inflammatory” therapy. Future clinical trials will be essential to define the safety, efficacy, and optimal patient populations for such strategies in the management of hypertension.

## 7 Cardiac arrhythmias

Atrial fibrillation (AF), the most common sustained arrhythmia, is increasingly recognised as a condition with a strong inflammatory component, where inflammasome-mediated pyroptosis plays a pivotal role in both electrical and structural atrial remodeling. Seminal work by Yao et al. demonstrated that NLRP3 inflammasome activation in atrial cardiomyocytes directly promotes AF by fostering ectopic firing, atrial fibrosis, and creating a substrate for sustained arrhythmia (88). Consistently, atrial biopsies from patients with chronic AF reveal increased expression of the active caspase-1 p20 subunit and higher levels of IL-1β and IL-18 in both tissue and serum compared to individuals in sinus rhythm, correlating with AF burden and persistence (9, 89). Mechanistic insights reveal that NLRP3 activation in atrial myocytes impairs calcium handling through CaMKII signaling and disrupts connexin-mediated conduction, thus promoting electrical remodeling and reentry circuits (90). GSDMD–mediated pyroptosis has been directly linked to arrhythmogenesis: enforced expression of the GSDMD N-terminal fragment in murine atria increased AF susceptibility, promoted IL-1β release, and triggered macrophage recruitment (91). Furthermore, epicardial adipose tissue-derived IL-1β, especially in post-cardiac surgery patients, has been causally implicated in triggering postoperative AF through promoting local inflammation and atrial fibrosis (92). Clinical studies further confirm that AF patients typically present with elevated inflammasome cytokines. Elevated IL-1β and IL-18 predict AF persistence or recurrence after ablation, and inflammatory markers such as CRP and IL-6 are associated with increased risk of AF, especially following cardiac surgery (89, 93). Importantly, factors such as oxidative stress, obesity, diabetes, aging, and gut dysbiosis can all prime or activate the NLRP3 inflammasome in atrial tissue, highlighting a convergence of metabolic and inflammatory risk (94–97).

**Figure 2:**
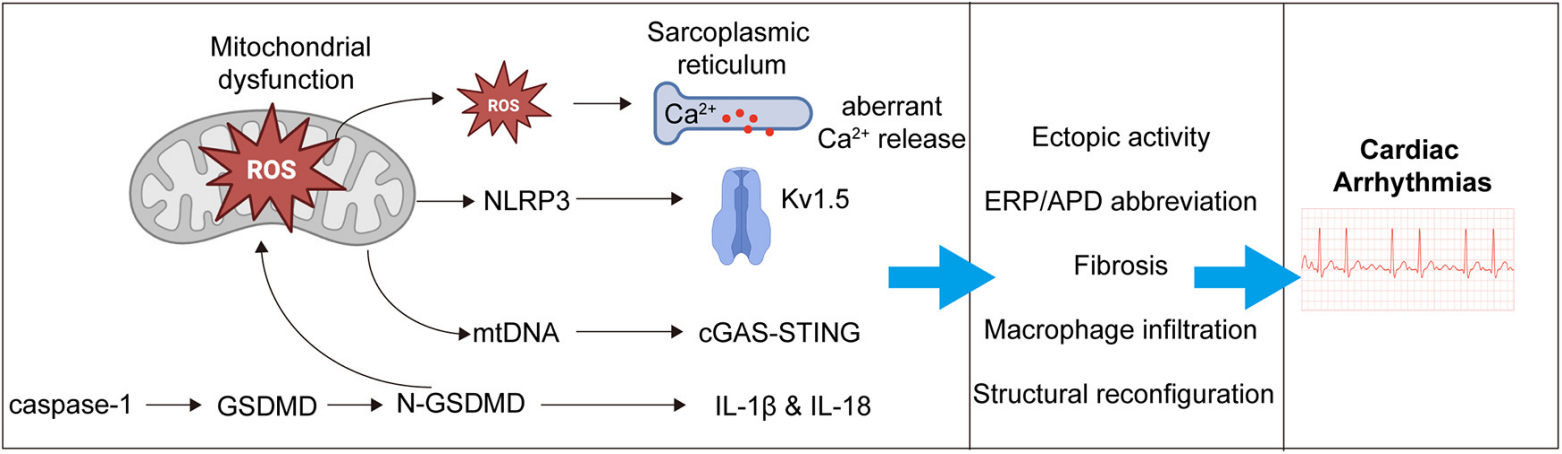
Mechanistic pathways linking pyroptosis to the development of atrial fibrillation. Activated gasdermin D (GSDMD) promotes the development of AF through both membrane pore formation and mitochondrial injury. Upon cleavage, the N-terminal fragment (NT-GSDMD) forms pores in the plasma membrane of atrial cardiomyocytes, facilitating the release of interleukin-1β (IL-1β), which triggers local inflammation, macrophage infiltration, and atrial fibrosis. Simultaneously, NT-GSDMD translocates to the mitochondrial membrane, leading to mitochondrial dysfunction and overproduction of reactive oxygen species (ROS). These ROS disrupt intracellular calcium homeostasis, enhance sarcoplasmic reticulum (SR) calcium release, and increase the risk of ectopic electrical activity. ROS also activate the NLRP3 inflammasome, upregulating potassium channels (e.g., Kv1.5), shortening action potential duration, and promoting atrial electrical remodeling. Additionally, mitochondrial damage causes the release of mitochondrial DNA (mtDNA), which activates the cyclic GMP-AMP synthase (cGAS)–stimulator of interferon genes (STING) pathway, further amplifying the inflammatory response. Together, these events drive structural remodeling, immune cell infiltration, and electrical instability, creating a pro-arrhythmic substrate for the initiation and maintenance of AF.

GSDMD plays a pivotal pathogenic role in the development of atrial fibrillation (AF). On one hand, it forms membrane pores in atrial cardiomyocytes, facilitating the release of interleukin-1β (IL-1β), thereby triggering local inflammatory responses, promoting atrial fibrosis, and recruiting macrophage infiltration (93, 98). On the other hand, NT-GSDMD anchors to the mitochondrial membrane, compromising its structural integrity and leading to mitochondrial dysfunction with excessive release of reactive oxygen species (ROS). Mitochondria-derived ROS disrupt intracellular calcium homeostasis, enhance sarcoplasmic reticulum calcium release, and increase the likelihood of ectopic electrical activity (99, 100). In addition, ROS activate the NLRP3 inflammasome pathway, which upregulates potassium channels (e.g., Kv1.5), shortens action potential duration in atrial myocytes, and contributes to atrial electrical remodeling. Moreover, mitochondrial damage results in the release of mitochondrial DNA (mtDNA), which activates the cyclic GMP-AMP synthase (cGAS)-stimulator of interferon genes (STING) pathway, further amplifying inflammatory responses (93). Collectively, these events promote atrial fibrosis, immune cell infiltration, and electrical instability, thereby providing a pathological substrate for the initiation and maintenance of atrial fibrillation (Figure 2).

**SCHEMATIC ILLUSTRATION:**
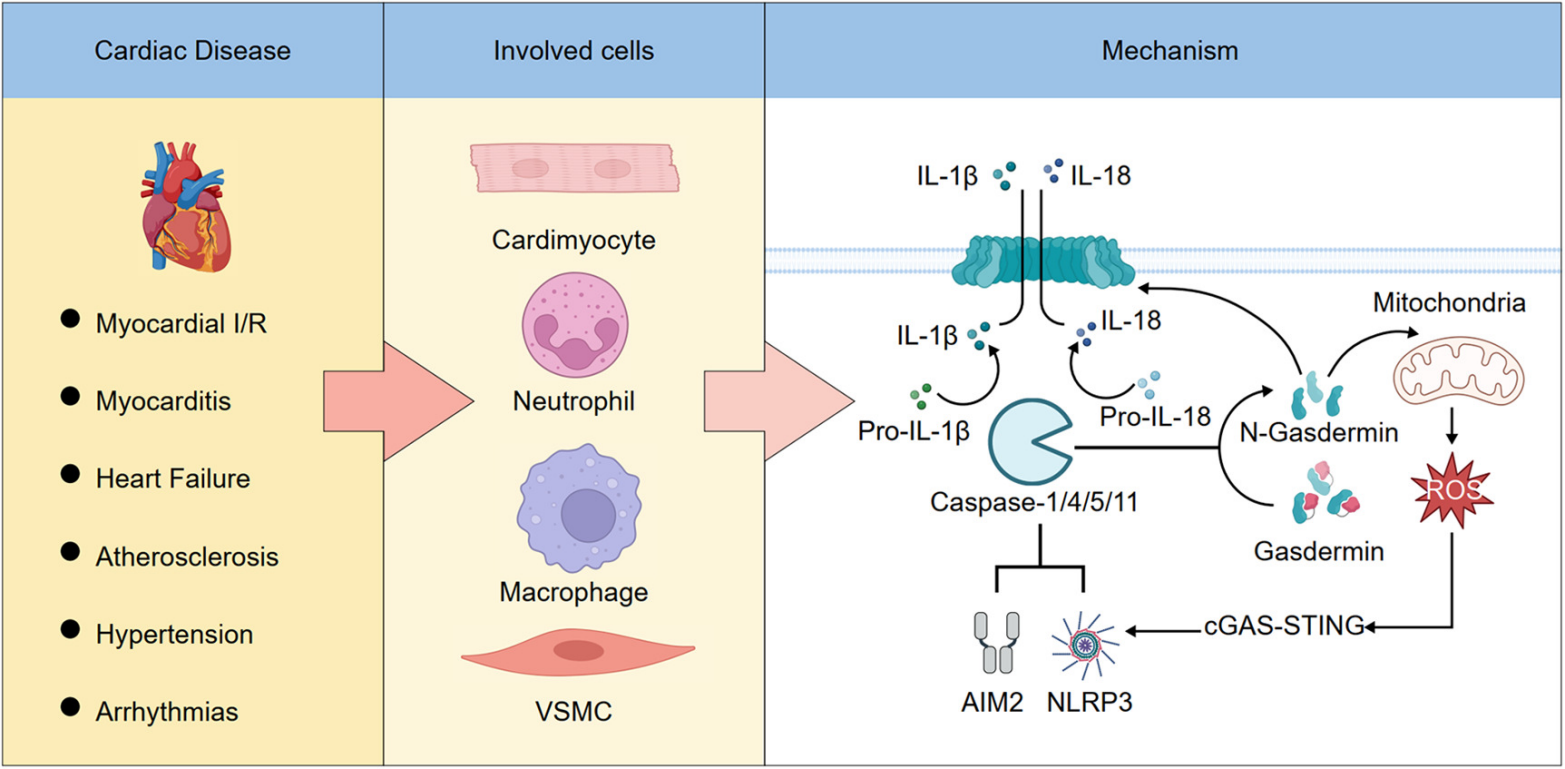
The role and mechanism of pyroptosis in cardiovascular diseases. (Left) Pyroptosis plays a pivotal role in various cardiovascular diseases. (Middle) It occurs in multiple cardiac cell types. (Right) Activation of the NLRP3 or AIM2 inflammasomes leads to the cleavage and activation of caspase-1, -4, -5, or -11. These activated caspases then process pro-inflammatory cytokines pro-IL-1β and pro-IL-18 into their mature forms, IL-1β and IL-18. In addition, caspase-1/4/5/11 cleave gasdermin D (GSDMD), releasing its N-terminal fragment, which forms pores in the plasma membrane and mitochondria. Pore formation on the plasma membrane allows IL-1β and IL-18 to be released into the extracellular space, leading to pyroptotic cell death. Mitochondrial pore formation increases the release of reactive oxygen species (ROS), which further amplifies inflammasome activation, forming a feed-forward loop.

### 7.1 Translational and therapeutic perspective

These insights into inflammasome–pyroptosis pathways offer promising therapeutic implications for arrhythmia management. Pharmacological inhibition of NLRP3 with compounds such as MCC950 has been shown to prevent electrical remodeling and reduce AF inducibility in animal models (88, 101, 102). In the clinical setting, anti-inflammatory agents like colchicine have demonstrated efficacy in reducing the incidence and recurrence of post-operative and post-ablation AF, as confirmed by meta-analyses (103, 104). Other interventions, including IL-1β blockers (e.g., canakinumab), statins, and RAAS inhibitors, have shown variable but generally protective effects, likely mediated through attenuation of upstream inflammatory or oxidative stress signaling (105–107). Notably, IL-1 blockade after AF cardioversion may reduce recurrence, though larger trials are needed (108). Lifestyle interventions such as weight loss, improved glycaemic control, and exercise may also blunt atrial inflammasome activation and thus reduce AF risk, supporting a holistic management paradigm. In patients with HF or HFpEF, targeting inflammasome signaling can also lower AF vulnerability and arrhythmic remodeling (102, 109). Collectively, mounting evidence indicates that inflammation, particularly via the NLRP3–caspase-1–GSDMD/IL-1β/IL-18 axis, is a central driver of both electrical and structural remodeling in AF. Targeting these pathways—pharmacologically or through upstream risk modification—represents a promising adjunct to conventional rhythm and rate control strategies, particularly in patients with high inflammatory burden or comorbid metabolic disease.

## 8 Conclusion

Multiple forms of cell death—including pyroptosis, apoptosis, necroptosis, and ferroptosis—contribute to the pathogenesis of cardiovascular diseases through distinct molecular mechanisms and cellular processes (110–116), as summarized in Table 1. Pyroptosis represents a pivotal inflammatory cell death pathway that bridges innate immune activation with irreversible cardiac and vascular injury. Increasing evidence highlights its substantial involvement in the initiation and progression of diverse CVDs, including MI, myocarditis, HF, atherosclerosis, hypertension, and arrhythmias. By amplifying local and systemic inflammation through gasdermin-mediated membrane rupture and cytokine release, pyroptosis drives adverse tissue remodelling and clinical deterioration. Recent experimental and early translational studies suggest that targeting key components of the pyroptotic machinery—such as NLRP3, caspases, and gasdermins—may attenuate organ damage and improve outcomes in CVDs. This is summarized in the Schematic Illustration, which outlines the role and mechanism of pyroptosis in cardiovascular diseases. Nonetheless, significant challenges remain, including the need for precise biomarkers, improved understanding of disease- and cell-specific roles, and the development of selective, safe inhibitors suitable for clinical application. Further research into the temporal and spatial regulation of pyroptosis and its interplay with other death modalities will be essential for translating these insights into effective therapies. Ultimately, modulating pyroptosis holds promise as a novel avenue for CVDs intervention and risk stratification.

Recent studies have highlighted pyroptosis-related molecules—particularly gasdermin D (GSDMD), interleukin-1β (IL-1β), and interleukin-18 (IL-18)—as potential biomarkers for cardiovascular diseases (111). Circulating GSDMD levels are elevated in patients with acute myocardial infarction and heart failure, correlating with infarct size and inflammatory cytokine profiles. Although ELISA kits for GSDMD, IL-1β, and IL-18 are available with some achieving clinical-grade sensitivity, large-scale, multicenter validation remains lacking (110). While IL-1β and IL-18 are broadly elevated across various inflammatory conditions, limiting their specificity for cardiovascular pathology, GSDMD's proximal role in the pyroptotic cascade may offer improved diagnostic precision (35). However, assay standardization and reference range establishment are urgently needed (34). Moreover, the dynamic temporal patterns of these biomarkers throughout disease onset, progression, and resolution are not yet fully characterized. Future prospective studies should aim to elucidate their diagnostic and prognostic utility and assess their integration with established cardiac biomarkers, such as troponins and B-type natriuretic peptide, to enhance cardiovascular risk stratification.

**Table 1:** Comparative characteristics of pyroptosis, apoptosis, necroptosis, and ferroptosis in cardiovascular diseases.

Cell death type	Key molecules	Morphological features	Inflammatory	Role in CVDs	Therapeutic targets
Pyroptosis	Caspase-1/4/5/11, GSDMD, GSDME, NLRP3	Cell swelling, membrane pore formation, lysis	Yes	Myocarditis, Myocardial infarction, Heart failure, Atherosclerosis, Arrhythmia	NLRP3 inhibitors (e.g., MCC950), caspase-1 inhibitors (e.g., VX-765), GSDMD blockers
Apoptosis	Caspase-3/7, Bcl-2 Bax	Cell shrinkage, chromatin condensation, membrane blebbing	No	Ischemia-reperfusion injury, Heart failure	Caspase inhibitors
Necroptosis	RIPK1/RIPK3, MLKL	Organelle swelling, membrane rupture	Yes	Ischemia/reperfusion injury, Heart failure, Diabetic cardiomyopathy	RIPK1 inhibitors (e.g., Nec-1) MLKL inhibitors
Ferroptosis	GPX4, ACSL4, lipid ROS Fe2^+^	Iron-dependent lipid peroxidation, mitochondrial shrinkage	Yes	Doxorubicin-induced cardiomyopathy, Heart failure, Atherosclerosis	Ferrostatins, iron chelators, GPX4 activators

Although extensive preclinical studies have demonstrated the pathogenic role of pyroptosis in cardiovascular diseases such as myocarditis, heart failure, and atrial fibrillation, the clinical translation of anti-pyroptotic therapies remains challenging. Key pathway inhibitors—including MCC950 (targeting the NLRP3 inflammasome) and VX-765 (a caspase-1 inhibitor)—have shown therapeutic potential in animal models but are limited by suboptimal pharmacokinetics, poor tissue specificity, and risks of immunosuppression and infection during long-term use. A study shows that while these agents can improve cardiac function and attenuate remodeling, their narrow therapeutic windows and potential to impair host immunity constrain their clinical applicability (117). Moreover, the degree of pyroptosis activation varies across patient populations and disease stages, underscoring the need for reliable biomarkers to guide patient stratification and individualized therapy. A recent review highlighted that circulating or tissue levels of IL-1β, IL-18, and cleaved GSDMD may serve as valuable diagnostic and prognostic indicators (44). Notably, pyroptosis often acts in concert with apoptosis and necroptosis via the PANoptosis pathway, indicating that single-target therapies may be insufficient to halt inflammation-driven tissue injury. Current strategies increasingly emphasize combination regimens that integrate anti-pyroptotic agents with anti-fibrotic or immunomodulatory therapies to enhance efficacy (44, 118). Importantly, the timing of intervention is critical—early-phase blockade may effectively reverse pathological remodeling, whereas late-stage inhibition may be less beneficial or even detrimental. Furthermore, obstacles such as limited access to cardiac tissue, a lack of standardized clinical endpoints, and insufficient mechanistic validation continue to impede the clinical development of anti-pyroptosis therapies.
