# Correction: Pyroptosis in cardiovascular diseases: roles, mechanisms, and clinical implications

**DOI:** 10.3389/fcvm.2025.1680298

**Published:** 2025-09-11

**Authors:** Yuqing Niu, Li Wang, Yaoqing Zhang, Yanqiang Zou, Cheng Zhou

**Affiliations:** ^1^ Department of Cardiovascular Surgery, Union Hospital, Tongji Medical College, Huazhong University of Science and Technology, Wuhan, China; ^2^ Department of Cardiovascular Surgery, Jingshan Union Hospital, Union Hospital, Huazhong University of Science and Technology, Jingshan, Hubei, China; ^3^ Department of Pharmacy, Qiaokou District People’s Hospital, Wuhan, China

**Keywords:** pyroptosis, cardiovascular diseases, myocardial infarction, myocarditis, heart failure, atherosclerosis, hypertension, arrhythmia

Affiliation Department of Cardiovascular Surgery, Union Hospital, Tongji Medical College, Huazhong University of Science and Technology, Wuhan, China was erroneously given as Department of Cardiovascular Surgery, Jingshan Union Hospital, Union Hospital, Huazhong University of Science and Technology, Jingshan, Hubei, China.

Affiliation Department of Cardiovascular Surgery, Jingshan Union Hospital, Union Hospital, Huazhong University of Science and Technology, Jingshan, Hubei, China was erroneously given as Department of Cardiovascular Surgery, Union Hospital, Tongji Medical College, Huazhong University of Science and Technology, Wuhan, China.

As such the authors affiliations have also been rectified. Author Yaoqing Zhang is affiliated to Department of Cardiovascular Surgery, Jingshan Union Hospital, Union Hospital, Huazhong University of Science and Technology, Jingshan, Hubei, China. Authors Yanqiang Zou and Cheng Zhou are affiliated to Department of Cardiovascular Surgery, Union Hospital, Tongji Medical College, Huazhong University of Science and Technology, Wuhan, China.

Authors Yuqing Niu, Li Wang and Yaoqing Zhang were erroneously omitted as equal contributing authors.

The original version of this article has been updated.

